# HAE therapies: past present and future

**DOI:** 10.1186/1710-1492-6-23

**Published:** 2010-07-28

**Authors:** Bruce L Zuraw

**Affiliations:** 1 Department of Medicine, University of California San Diego and San Diego Veteran's Affairs Medical Center, La Jolla, CA, USA

## Abstract

Advances in understanding the pathophysiology and mechanism of swelling in hereditary angioedema (HAE) has resulted in the development of multiple new drugs for the acute and prophylactic treatment of patients with HAE. This review will recap the past treatment options, review the new current treatment options, and discuss potential future treatment options for patients with HAE.

## Introduction

Hereditary angioedema with reduced C1 inhibitor function (HAE) is an autosomal dominant disease characterized by recurrent episodes of potentially life-threatening angioedema. The pathophysiology of HAE as well as the molecular mechanisms underlying attacks of swelling in HAE have been gradually dissected over the past 50 years [1-3]. These advances have led to a rapidly changing set of therapeutic options for patients with HAE.

HAE patients typically begin to swell in childhood, and often suffer increased symptoms about the time of puberty, and continue to experience recurrent attacks of angioedema throughout the remainder of their lives [4]. Attacks of angioedema in HAE can be severe and prolonged, typically lasting 3-5 days before the patient is well again. Abdominal attacks may result in hospitalization and all to often lead to inappropriate intra-abdominal surgery, while oro-pharyngeal-laryngeal attacks can be life-threatening [4-6]. Despite striking advances in medical knowledge, HAE patients continue to die from laryngeal attacks [7,8]. The disease thus imposes an enormous burden on patients as well as their families, often preventing them from leading a productive life.

Because of the significant morbidity and mortality associated with HAE, careful management of these patients is essential. The management of HAE required attention to three areas: treatment of acute episodes of angioedema, long-term prophylaxis, and short-term prophylaxis [4,5,9,10]. To help the clinician navigate the changing therapeutic landscape, this article will review the past, current, and future options for treating HAE patients in the United States.

### HAE treatment: The past

#### Treatment of acute HAE attacks

Attacks of angioedema in patients with HAE involve subcutaneous tissues (primarily involving extremities, genitalia or the face), the intestine, and the respiratory tract. Attacks typically but not invariably follow a trajectory in which the angioedema increases for 24 hours then slowly decreases over the following 48-72 hours. Importantly, the swelling in HAE attacks does not respond reliably to the drugs employed in treating other forms of urticaria/angioedema such as anti-histamines, epinephrine, or corticosteroids. While epinephrine, in particular, may have a transient effect on swelling, it does not alter the course of the attack.

Until late 2008, there was no drug approved in the United States that was predictably effective for the treatment of acute attacks of HAE. Anecdotal and published experience suggests that administration of fresh frozen plasma can abort ongoing HAE attacks by replacing plasma C1 inhibitor (C1INH) levels [11]. There is, however, a theoretic and demonstrated risk that fresh frozen plasma can worsen acute swelling, possibly due to replenishment of plasma proteases and substrates involved in the generation of peptides that mediate the angioedema [12,13]. Epsilon aminocaproic acid (Amicar™) has also been used intravenously for acute episodes of angioedema, and anecdotal reports suggest that it may be minimally helpful; however, there is no published evidence demonstrating that it provides significant benefit. Anabolic androgens, which are effective prophylactic agents (see below) require at least 1-2 days before they begin to be effective, and are therefore not useful in the acute treatment of attacks.

The management of acute attacks was thus primarily concerned with symptomatic control of the swelling. Abdominal attacks often present with severe pain and nausea as well as significant dehydration, sometimes accompanied by significant hypotension. Management of these attacks involved aggressive intravenous replacement of fluid as well as control of pain and nausea with parenteral narcotic and antiemetic drugs. Oropharyngeal attacks may lead to death secondary to asphyxiation, and therefore required hospitalization for careful monitoring of airway patency. If the airway was threatened, the patient needed to be intubated by an experienced physician with the capability for emergency tracheotomy immediately available. Acute angioedema of the extremities does not typically require treatment, although angioedema of the feet or dominant hand can be temporarily disabling.

#### Long-term prophylaxis

The goal of long-term prophylaxis is to decrease the frequency and/or severity of swelling attacks. The frequency and severity of angioedema attacks is highly variable among HAE patients, ranging from attacks occurring as often as twice per week to patients who are asymptomatic. Most untreated HAE patients will swell approximately one to two times per month on average. While some HAE patients may not require long-term prophylactic therapy, patients with frequent attacks or with a history of serious attacks involving the upper airway should be treated prophylactically. In general patients with significant swelling occurring more frequently than once every 3 months are considered candidates for long-term prophylactic therapy, although it is the impact of the episodes on the patient's ability to lead a normal life that is the deciding factor. Other considerations that should go into this decision include the location of attacks (airway attacks causing increased concern) and the accessibility of the patient to appropriate medical care. Because of their ability to increase bradykinin-mediated effects, angiotensin-converting enzyme inhibitors need to be avoided in HAE patients. Birth control pills and hormonal replacement therapy also frequently exacerbate disease severity in women [14].

Two modalities of treatment were available for long-term prophylaxis: anabolic androgens and anti-fibrinolytics. The best tolerated and most effective long-term prophylactic drugs are the synthetic anabolic androgens which increase C1INH plasma levels and decrease attacks of HAE [15]. The 17-α-alkylated androgens are orally available and were the drugs of choice for the long-term prophylaxis of HAE. Danazol and stanozolol are synthetic 17-α-alkylated androgens that are widely used for this purpose and are less virulizing than methyltestosterone. Oxandrolone, a 17-α-alkylated androgen that is approved for treatment of acquired immunodeficiency syndrome wasting syndrome in children, has also been successfully used to treat HAE [16]. The precise mechanism by which anabolic androgens increase C1INH levels has not be elucidated [17]; but the dose of anabolic androgen should not be based on the C1INH response. The dose of anabolic androgens used to treat HAE should be titrated down to find the lowest dose which confers adequate prophylaxis, typically 2 mg stanozolol daily or every other day or 200 mg danazol daily or every other day. Detailed recommendations for dose titration have been published [18].

The side effects of anabolic androgens are dose related, with the most important side effects being hepatotoxicity and virulization [19]. Most HAE patients tolerate anabolic androgens at the doses described above, however sustained use at higher doses often result in significant side effects. Patients taking anabolic androgens should have their liver enzymes checked every 6 months. Evidence of hepatic injury should precipitate tapering or discontinuation of the drug, with documentation of normalization of the hepatic tests. Since hepatic adenomas have been reported as a consequence of anabolic androgens [20], ultrasound examination of the liver is warranted in the presence of persistently elevated liver enzymes.

The antifibrinolytic drugs epsilon aminocaproic acid (EACA or Amicar) and tranexamic acid are frequently but not always effective in preventing angioedema attacks in HAE [21-23]. The mechanism of their efficacy in HAE is unknown. Because the anabolic androgens are more reliably effective for the control of HAE, they were generally used in preference to antifibrinolytics in adult patients with the antifibrinolytic drugs often reserved for patients who didn't tolerate anabolic androgens. Because anabolic androgens may interfere with normal sexual maturation, antifibrinolytics have been preferred over androgens in children and pregnant women. Tranexamic acid is not currently available in the United States. The typical therapeutic dose of EACA is 1 gm orally 3-4 times per day.

The treatment of pregnant women and children presented particular difficulties. Androgens are contraindicated in these populations due to their potential effects on growth and sexual maturation. Angioedema frequency may not change or may decrease during pregnancy; however, some women experience an increase in attacks during pregnancy. Remarkably, almost all women are protected from swelling during labor and delivery.

#### Short-term prophylaxis

Short-term prophylaxis should be used to prevent attacks of angioedema when the patient is at high risk of swelling, particularly before expected trauma such as surgery or dental procedures. To avoid potentially catastrophic swelling, it is critically important that all HAE patients be made aware of the need for short-term prophylaxis in these situations.

High-dose anabolic androgen therapy (stanozolol 2 mg three times daily or danazol 200 mg three times daily) begun 5 to 7 days before the procedure affords reasonable protection in most patients [18]. Alternatively, the patient can be infused with two units of fresh frozen plasma several hours before the procedure [24].

### HAE treatment: The present

Over the past 18 months, 3 new medications for the treatment of HAE have been approved for use in the United States. Two of these medications are C1INH concentrates and the third is a plasma kallikrein inhibitor. Each of these is discussed below.

#### Plasma-derived C1INH concentrates

The pathophysiologic basis of HAE was demonstrated to be a deficiency of C1INH in 1963 by Virginia Donaldson [1], clarifying the lack of kallikrein inhibitory activity in HAE patient plasma observed the year before by Landerman et al [25]. The rationale for replacement therapy was established by the success of administering fresh frozen plasma (FFP) during acute attacks of HAE [11]. Beginning in the late 1970s, a number of investigators in Europe and the United States began demonstrating that replacement therapy with C1INH concentrates was effective in HAE.

Over the past 25 years, multiple studies have confirmed the efficacy of plasma C1INH as replacement therapy for acute attacks of HAE [26-32]. Clinically, symptomatic improvement is typically seen within 30-60 minutes of drug administration [33]. Furthermore, C1INH concentrates appear to be equal efficacy for all types of HAE attacks - including laryngeal attacks where it can be life-saving [31]. C1 inhibitor concentrates have also been successfully used for both short-term [34-37] and long-term prophylactic treatment of HAE [38-40]. C1INH concentrate became the preferred modality of treatment for acute attacks of HAE in some countries where it is available.

In 1996, Waytes et al [41] published the results of two double-blind placebo-controlled studies comparing plasma derived C1INH (25 plasma units/kg; Immuno AG) to placebo. The first was a crossover study involving prophylactic treatment of 6 severely affected HAE patients who received study drug every three days. During the periods that they received C1INH, subjects increased their plasma C1INH functional levels, normalized their C4 titers and had significantly less swelling than they did during the period they received placebo. The second study assessed the time to improvement following study drug in 22 patients with acute attacks of HAE. The beginning of relief occurred significantly faster in C1INH treated patients than in placebo treated patients (55 versus 563 minutes). However, a pivotal phase III trial of the Immuno C1INH concentrate (Baxter Healthcare) for acute HAE attacks failed to show any improvement in C1INH-treated compared to placebo-treated subjects. Two plasma-derived C1INH products underwent Phase 3 randomized clinical trials, and were recently approved for use in the United States.

#### Pasteurized plasma-derived C1INH concentrate

Berinert (CSL Behring) is a pasteurized lyophilized human plasma-derived C1 inhibitor concentrate for intravenous injection. It has been licensed in Europe (Germany, Austria, and Switzerland) for over 20 years, and is also available in Canada. Numerous reports of the efficacy and safety of Berinert have been published (reviewed in [39]). A phase III study of Berinert for the treatment of acute attacks of HAE was recently completed [42]. This study compared the efficacy (shortening onset of relief of symptoms) of 2 doses of Berinert (10 U/kg and 20 U/kg) to placebo in 125 HAE patients with moderate to severe abdominal or facial angioedema attacks. Compared to the placebo treated group, subjects receiving 20 U/kg of Berinert-P showed a significant reduction in the median time to onset of relief of symptoms of HAE attacks compared to placebo (0.5 versus 1.5 hours, p = 0.0025). Median time to complete resolution of all HAE symptoms was also significantly shorter in the 20 U/kg group compared to the control group (4.92 versus 7.79 hours, p = 0.0237). At a dose of 10 U/kg, the median time to onset of relief was 1.2 hours, which was not significantly different than the placebo group.

Based on the data from this study, Berinert received approval from the FDA for use in the treatment of acute angioedema attacks in adolescent and adult HAE patients.

#### Nanofiltered and pasteurized plasma-derived C1INH concentrate

Cinryze (ViroPharma Incorporated) is a nanofiltered pasteurized C1INH concentrate for intravenous use. Cinryze is manufactured by Sanguin in the Netherlands, using U.S. plasma. The manufacturing process is identical to that used for the existing Cetor C1INH product, except that Cinryze is subjected to a final nanofiltration step, which provides additional protection against enveloped and non-enveloped viral particles and possibly prions [43]. Two separate randomized double-blind placebo controlled studies of Cinryze have been performed in the United States [44].

The first study assessed efficacy and safety of C1INH-nf for the treatment of moderate to severe acute attacks of facial, abdominal or genitourinary angioedema in HAE patients [45]. Subjects were infused with study drug (C1INH-nf 1,000 IU or placebo) at time 0. If significant relief was not reported within 60 minutes, subjects were then given a second dose of the same study drug they received initially. All subjects were eligible to receive open-label Cinryze after 4 hours. In 68 randomized eligible attacks, the estimated time to beginning of unequivocal relief (primary endpoint) was significantly shorter in the C1INH group (median time 2 hours) than in the placebo group (median time > 4 hours) (p = 0.026). Cinryze treated patients also showed a statistically significant improvement in median time to complete resolution of the defining symptoms (p = 0.004). The efficacy of Cinryze treatment did not vary by attack location.

A second study involved the use of C1INH-nf as long-term prophylaxis to prevent attacks of angioedema was also recently completed. Twenty-two patients with a history of frequent angioedema were treated with C1INH-nf (1,000 IU) or placebo two times per week for 12 weeks then crossed over and received the other treatment for an additional 12 weeks. During the C1INH-nf treatment periods, subjects showed a highly significant (p < 0.0001) decrease in HAE attacks (6.26 versus 12.73 attacks; p < 0.0001).

Cinryze received FDA approval for prophylactic treatment in adolescent and adult HAE patients. The application for use of Cinryze to treat acute attacks of angioedema is still pending.

#### Safety and tolerability of plasma-derived C1INH concentrates

Both Berinert and Cinryze are each derived from U.S. plasma that has been PCR screened then subjected to multiple viral inactivation/removal steps, including pasteurization. In addition, Cinryze undergoes nanofiltration, which removes viral- and potentially prion-sized particles based on size exclusion rather than specific physicochemical interactions. The results of the studies described above did not show any evidence of safety or tolerability issues with either of the drugs.

#### Plasma kallikrein inhibitor: ecallantide

Unraveling the mechanism of swelling in patients with HAE has long been considered central to the development of more effective treatment strategies. Early investigations found that incubation of plasma from HAE patients *ex vivo *at 37°C generated a factor that caused smooth muscle contraction and increased vascular permeability [46]. This 'vascular permeability enhancing factor' was correctly assumed to be the mediator of swelling in HAE; however, the final characterization of the factor remained elusive and controversial for many years. Compelling laboratory and clinical data have conclusively shown that bradykinin is the primary mediator of swelling in HAE [47-57]. The nanopeptide bradykinin is generated when active plasma kallikrein cleaves high molecular weight kininogen (HMWK) [58]. The released bradykinin moiety potently increases vascular permeability by binding to its cognate receptor (the bradykinin B2 receptor) on vascular endothelial cells.

The discovery that bradykinin is primarily responsible for the attacks of swelling in HAE has led to new therapeutic strategies to treat HAE by preventing bradykinin-mediated enhancement in vascular permeability. Replacement therapy with C1INH will inhibit both plasma kallikrein and activated factor XII. Indeed administration of C1INH concentrate has been shown to acutely reduce bradykinin levels in patients experiencing angioedema attacks [53]. Inhibition of plasma kallikrein using other non-C1INH drugs is another strategy that has been used. The first plasma kallikrein inhibitor, other than C1INH, to be used for the treatment of HAE was aprotinin (Trasylol^®^). This protein is a broad-spectrum Kunitz-type serpin inhibitor with activity against trypsin, plasmin and plasma kallikrein. While aprotinin was effective in halting acute attacks of HAE [26,59], this bovine protein was associated with severe anaphylactic reactions which precluded its use in HAE management [60,61]. More recently, a specific plasma kallikrein inhibitor, ecallantide, has been developed.

Ecallantide (Kalbitor, Dyax Inc.) is a novel, potent and specific plasma kallikrein inhibitor produced in the *Pichia pastoris *strain of yeast that was identified using phage display technology for a library of rationally designed variants of the first Kunitz domain of human lipoprotein-associated coagulation inhibitor (LACI) [62,63]. The recommended dose of ecallantide to treat an angioedema attack is 30 mg, administered as three 1 ml subcutaneous injections. Maximum ecallantide levels are reached 2-3 hours following subcutaneous injection, and the half-life is approximately 2 hours [64].

Two separate RDBPC phase III studies of ecallantide for the treatment of acute attacks of HAE have been performed in the United States. Both studies involved subjects randomized 1:1 to receive either ecallantide 30 mg or placebo by subcutaneous injection during a moderate or worse attack at any location. The first trial (EDEMA3) consisted of 72 patients with the primary endpoint measured as a treatment outcome score (TOS) at 4 hours. TOS is a patient-reported measure of response to therapy using a categorical scale from 100 (significant improvement) to -100 (significant worsening) for each symptom complex, weighted according to its baseline severity. Ecallantide-treated patients reported a mean TOS score of 49.5 ± 59.4 compared to 18.5 ± 67.8 in placebo-treated patients (p = 0.037) [65]. The improvement in TOS score was maintained at 24 hours (44.3 ± 70.4 versus -0.5 ± 87.9, p = 0.044).

The second trial (EDEMA4) consisted of 96 patients with the primary endpoint being mean symptom complex severity (MSCS) at 4 hours. The MSCS score is a patient-reported point-in-time measure of symptom severity based on symptom rating of 0 (none) to 3 (severe) for each potential symptom complex. Severity at each time point is the average across all symptom complexes. Ecallantide-treated subjects reported a mean decrease in symptom score at 4 hours of 0.81 compared to a decrease of 0.37 in placebo-treated subjects (p = 0.01). At 24 hours, mean symptom scores fell by 1.5 in the ecallantide-treated subjects compared to 1.1 in the placebo-treated subjects (p = 0.039).

No differences were observed in the response to ecallantide based on the location of swelling; however subjects who presented relatively late in the attack (6-8 hours) showed less benefit than those who presented earlier [66].

Safety is always paramount during drug development and some concerns have arisen regarding the use of ecallantide. Prolongation of the aPTT is commonly seen, without any enhanced risk of bleeding. Anaphylactic-like reactions have been reported in some subjects following exposure to ecallantide, including one subject who experienced a repeat reaction on re-challenge. A single first dose anaphylactic-like reaction to ecallantide described serum antibodies to a low molecular component of the drug, detected by immunoblotting [67]. Controversy remains as no antibodies were detected by ELISA screening performed by the manufacturer [68]. A proportion of patients who receive repeated injections of ecallantide will develop anti-drug antibodies. A relationship between the presence of anti-drug antibodies and risk of anaphylactoid reactions has yet to be observed, and many of the antibody positive subjects have continued to use ecallantide with good results.

Based on data from both Phase III studies [69], approval for use of ecallantide to treat acute HAE attacks in patients aged 16 and over was granted on December 2 2009. Because of the safety concerns reviewed above, there is a black box warning on anaphylactic potential and requiring that the drug be administered by a health care provider.

#### Summary of current therapeutic options

The approval of Berinert, Cinryze and ecallantide has completely changed the therapeutic options available for the treatment of HAE in the United States. Berinert and ecallantide are approved for treatment of acute attacks of angioedema in HAE. These are the first drugs that are reliably effective for the acute treatment of HAE attacks. While it may be tempting to limit the use of these drugs to severe or life-threatening attacks, it is clear that their efficacy is highest when they are used early in an attack when it is impossible to predict which attacks are likely to become severe or life-threatening. In all likelihood, therefore, these drugs will become the treatment of choice for acute attacks of angioedema in HAE patients. Long-term prophylaxis will still be important to limit the number of attacks needing acute treatment.

Cinryze is approved for HAE prophylaxis rather than acute treatment. In general, patients with relatively severe (≥ 2 attacks per month) HAE are potential candidates for prophylactic treatment with Cinryze. While significantly better than placebo, routine prophylaxis with Cinryze did not completely abrogate breakthrough attacks, and it is likely that individualization of the Cinryze dose or frequency of administration will be necessary to achieve optimal responses in all treated patients. It is also likely that low dose anabolic androgen therapy will continue to be useful in patients who tolerate these drugs.

### HAE treatment: The future

Two additional novel medications have undergone clinical trials and are potentially in the pipeline for use to treat acute attacks of angioedema in HAE patients.

#### Recombinant human C1INH

Rhucin (Pharming NV) is a recombinant human C1 inhibitor (rhC1INH) concentrate for intravenous infusion isolated from the milk of transgenic rabbits. It is identical to human plasma derived C1INH at the amino acid level and demonstrates the same inhibitory profile as plasma derived C1INH. However, rhC1INH has post-translational glycosylation differences compared to the plasma-derived product [70]. A phase I study of rhC1INH in which the drug was administered to 12 asymptomatic HAE patients at doses ranging from 6.25 to 100 U/kg [71] demonstrated a rapid increase in functional plasma C1INH activity and a corresponding fall in C4 activation, followed by a slower increase in C4 levels. The half-life of the protein was dose dependent and was longest at the highest dose used (100 U/kg) where it was estimated to be 3 hours. The accelerated clearance of rhC1INH from the plasma space compared to plasma derived C1INH was presumably influenced by the glycosylation differences in the recombinant protein. An open-label phase II study of rhC1INH demonstrated beginning of relief on average within 1 hour (median time 30 minutes), with time to minimal symptoms on average between 6 to 12 hours following infusion, and no evidence of late angioedema relapses [72].

Two separate phase III studies have been performed for rhC1INH in the treatment of acute attacks of angioedema in HAE patients http://www.pharming.com. A European randomized placebo-controlled double-blind clinical study of rhC1INH (100 U/kg) in 32 HAE patients was stopped on ethical grounds because of a strong and highly significant positive advantage for rhC1INH versus placebo in median time to beginning of relief (62 versus 508 minutes, p = 0.0009) as well as time to minimal symptoms (480 versus 1480 minutes, p = 0.0038).

The phase III study of rhC1INH (100 U/kg and 50 U/kg) in the United States and Canada in 39 HAE subjects showed a significant benefit for rhC1INH versus placebo in median time to beginning of relief (68 minutes for rhC1INH 100 U/kg, 122 minutes for rhC1INH 50 U/kg, and 258 minutes for placebo). Time to minimal symptoms was also significantly shortened after treatment with rhC1INH (245 minutes at 100 U/kg and 247 minutes at 50 U/kg) compared to placebo (1101 minutes).

There were no significant safety or tolerability issues reported in these phase III studies. One subject in an earlier phase study failed to report that she was allergic to rabbits, and experienced hives and wheezing after receiving rhC1INH

#### Icatibant

Another approach to treating HAE is by inhibiting the ability of bradykinin to bind to and signal through its cognate receptor, the bradykinin B2 receptor. In the C1INH knockout mouse, blockade of the biologic action of bradykinin using a bradykinin B2 receptor antagonist abolished the increased vascular permeability and provided proof of concept that bradykinin was the mediator of angioedema [57]. Lung et al [73] reported that HAE clinical severity was influenced by a polymorphism in the non-coding first exon of the bradykinin B2 receptor that impacted bradykinin B2 receptor expression. A recent report suggested that the permeability enhancement in HAE attacks may be transduced by the combination of bradykinin B2 receptors and bradykinin B1 receptors [74]; and thus, bradykinin antagonists that block both bradykinin receptors may have important advantages to just blocking the bradykinin B2 receptor.

Icatibant (Firazyr, Shire) is a synthetic selective decapeptide bradykinin B2 receptor competitive antagonist that contains five non-natural amino acids to enhance resistance to peptidases [75,76]. Icatibant is administered subcutaneously as a single 30 mg injection, achieves peak concentration within 30 minutes, and has a half-life of approximately 1-2 hours [77,78].

The safety and efficacy of icatibant for the treatment of acute HAE attacks was assessed in two RDBPC phase III studies [79]. One study compared icatibant to placebo in 56 subjects in the United States, Argentina, Australia and Canada (FAST-1). The other study compared icatibant to tranexamic acid in 72 subjects in Europe and Israel (FAST-2). Both studies involved subjects randomized 1:1 to receive either icatibant 30 mg by subcutaneous injection versus placebo (FAST1) or tranexamic acid (FAST2) during a moderate to severe abdominal or cutaneous angioedema attack. Primary endpoint was time to onset of symptom relief assessed by subject-recorded visual analog scale (VAS).

In the FAST-2 study, time to onset of relief was significantly faster in the icatibant treated subjects (2 versus 12 hours, p < 0.0001). Based on this, the drug was approved for use for acute attacks in the European Union. In contrast, the FAST-1 study failed to show a significant benefit for icatibant (2.5 versus 4.6 hours, p = 0.13). The FDA disapproved the application for licensure, and a new RDBPC phase III trial is ongoing.

Post-hoc analysis of the FAST-1 data suggests that this study did not reach statistical significance due to the confounding effect of narcotic pain relief given primarily to placebo patients for abdominal attacks. Icatibant was generally well tolerated. The most common side effect attributable to the drug was transient local pain and swelling at the site of injection. Additional attractive features of icatibant include its stability at room temperature and a shelf life of at least one year.

#### Other future directions

Several additional treatment options will be briefly mentioned. First, the possibility of administering C1INH concentrate by sub-cutaneous infusion is under active consideration. This route may be ideal for obtaining relatively steady plasma levels of C1INH during long-term prophylaxis. Second, the possibility that coagulation factor XII could become a therapeutic target. Like strategies targeting plasma kallikrein, inhibition of factor XII activity might prevent bradykinin generation [80]. Third, there is a possibility of developing orally available bradykinin receptor antagonists. Fourth, the recent demonstration that the bradykinin B1 receptor may play a role in the swelling of HAE patients [74] suggests the possibility of combined bradykinin B2 and B1 receptor antagonism may be more effective than antagonizing the bradykinin B2 receptor alone. Finally, advances in gene repair or intracellular trafficking may eventually open avenues for molecular correction of the defects in HAE.

## Conclusion

The treatment of HAE, after remaining static for nearly 40 years, has undergone rapid change during the past several years; and additional drugs are likely to be approved within the next several years.

Since the time to complete resolution of an acute attack is strongly influenced by the interval between symptom onset and institution of effective therapy [81], early self-treatment of acute attacks may provide the best way to minimize morbidity from breakthrough HAE attacks. The ease of use, stability and safety of icatibant are positive attributes that enhance the likelihood that it could be self-administered. While ecallantide is also administered by the subcutaneous route, the restrictions requiring administration by a health care professional would preclude self administration at this time.

Variability in attack frequency and severity, response to individual therapeutic agents, and the factors of gender, age, pregnancy, co-existing medical conditions, or access to medical care highlight the need for individualization in the approach to treatment of HAE. Ultimately, the introduction of these drugs coupled with the availability of C1 inhibitor will allow for a menu of options to incorporate into patient-centric treatment plans for HAE.

## Abbreviations

HAE: hereditary angioedema; EACA: epsilon aminocaproic acid; FFP: fresh frozen plasma; HMWK: high molecular weight kininogen; LACI: lipoprotein associated coagulation inhibitor; VAS: visual analog scale; MSCS: mean symptom complex severity; TOS: treatment outcome score; rhC1INH: recombinant human C1 inhibitor; C1INH: C1 inhibitor

## Competing interests

The author has been an investigator for HAE studies with Lev Pharmaceuticals, Dyax, Pharming, and Shire. He has been a consultant to Lev, ViroPharma, Dyax, Pharming, CSL Behring, Jerini, and Shire
